# Some conditions apply: Systems for studying *Plasmodium falciparum* protein function

**DOI:** 10.1371/journal.ppat.1009442

**Published:** 2021-04-22

**Authors:** Heather M. Kudyba, David W. Cobb, Joel Vega-Rodríguez, Vasant Muralidharan

**Affiliations:** 1 Laboratory of Malaria and Vector Research, National Institute of Allergy and Infectious Diseases, National Institutes of Health, Bethesda, Maryland, United States of America; 2 Department of Cellular Biology, University of Georgia, Athens, Georgia, United States of America; 3 Center for Tropical and Emerging Global Diseases, University of Georgia, Athens, Georgia, United States of America; Boston College, UNITED STATES

## Abstract

Malaria, caused by infection with *Plasmodium* parasites, remains a significant global health concern. For decades, genetic intractability and limited tools hindered our ability to study essential proteins and pathways in *Plasmodium falciparum*, the parasite associated with the most severe malaria cases. However, recent years have seen major leaps forward in the ability to genetically manipulate *P*. *falciparum* parasites and conditionally control protein expression/function. The conditional knockdown systems used in *P*. *falciparum* target all 3 components of the central dogma, allowing researchers to conditionally control gene expression, translation, and protein function. Here, we review some of the common knockdown systems that have been adapted or developed for use in *P*. *falciparum*. Much of the work done using conditional knockdown approaches has been performed in asexual, blood-stage parasites, but we also highlight their uses in other parts of the life cycle and discuss new ways of applying these systems outside of the intraerythrocytic stages. With the use of these tools, the field’s understanding of parasite biology is ever increasing, and promising new pathways for antimalarial drug development are being discovered.

## Introduction

Malaria remains a leading cause of morbidity and mortality worldwide, with approximately 228 million cases and over 400,000 deaths in 2018 [1]. The intracellular, eukaryotic parasite *Plasmodium falciparum* is responsible for the most severe cases of malaria. While significant progress has been made to drive down malaria cases and deaths, progress has plateaued in recent years due to the rise of *P*. *falciparum* parasites that are resistant to antimalarial drugs and development of insecticide resistance by the mosquito [1]. There is a constant need for new insights into druggable targets in *P*. *falciparum* biology, and therefore, a need for molecular tools to study essential proteins and pathways that support parasite survival. The *P*. *falciparum* genome is haploid for much of its life cycle, thus preventing the generation of random mutants except under specific scenarios (e.g., supplementing parasites with isopentenyl pyrophosphate to bypass the essential metabolic function of the apicoplast to study plastid-related genes) [2]. This precludes the use of random mutagenesis screens to identify biological mechanisms behind parasite-specific pathways in most cases. Furthermore, the *Plasmodium* parasite is evolutionarily distant from well-studied model organisms, and a large proportion of parasite genes lack homology outside of the phylum. This suggests that while some protein functions are conserved, their biological context is the parasitic life cycle of *Plasmodium* spp., which often prevents a direct comparison with what is known about the specific biological function of conserved proteins from work on model systems. The development and implementation of conditional gene or protein expression systems that allows spatial and temporal perturbation of protein function have helped overcome these hurdles. Further, an important technological advancement in studying essential genes in *P*. *falciparum* was the implementation of CRISPR/Cas-9 gene editing, which has allowed these conditional tools to be widely applied in *P*. *falciparum*.

**Fig 1:**
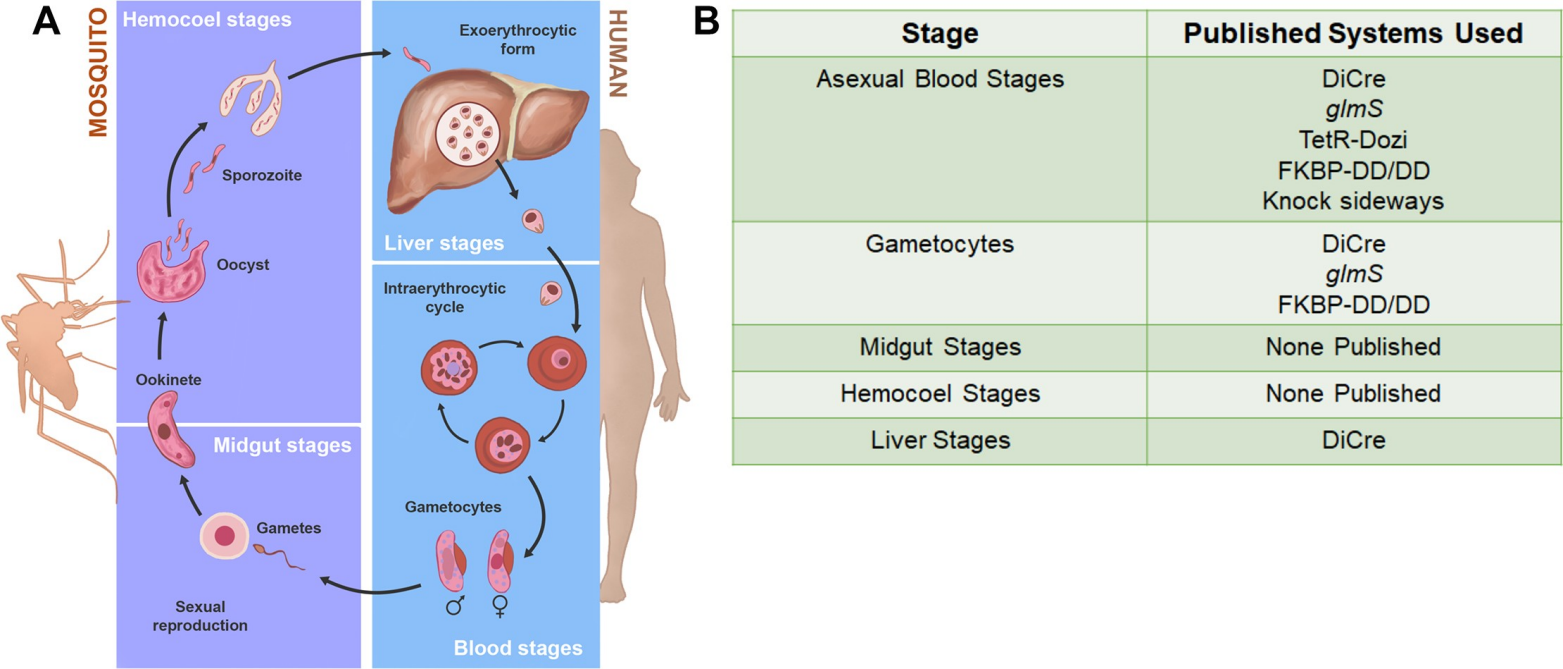
Conditional protein knockdown used throughout the *Plasmodium falciparum* life cycle. **(A)** Schematic of the malaria life cycle in the human host and the mosquito vector. **(B)** Table outlining the conditional knockout/knockdown systems used at various life cycle stages of *P*. *falciparum*.

In this review, we describe some of the conditional methods for knocking out or knocking down protein expression in *P*. *falciparum*. The ability to conditionally disrupt essential protein function is critical for understanding parasite biology and identifying new drug targets. Conditional systems exist at all levels of the central dogma of molecular biology, allowing researchers to modify the parasite genome, disrupt translation, and directly inhibit protein function based on the addition or removal of small molecules. Some of these systems have been adapted from use in other organisms—such as the Cre recombinase—and others have been developed specifically for *Plasmodium* research—such as the *Pf*DOZI-TetR aptamer system. With a few exceptions, these systems have been most widely used to study the blood stages of *P*. *falciparum*, which cause the clinical manifestations of malaria (**Fig 1**). This is due to the fact that the blood stages are relatively easy to grow and do not involve a metazoan host like the mosquito or a nucleated host cell like primary liver hepatocytes. However, the sexual and extraerythrocytic stages offer critical points for therapeutic intervention aiming at blocking malaria transmission, and we will also discuss the use of conditional systems outside of asexual parasites [3].

### Conditional gene knockout

One approach for conditional knockdown of transcription involves complete removal of DNA sequences from the genome by activating a site-specific recombinase expressed in the parasite. A major advantage of this approach is that entire genes can be removed, completely preventing transcription of those genes in parasites that successfully excise the DNA, although the knockout may not be 100% penetrant within the population. The use of site-specific recombinases in model organisms like *Drosophila melanogaster* and *Caenorhabditis elegans* has proven the technology powerful for studying gene function. Within the last decade, the use of site-specific recombinases in *P*. *falciparum* has become a powerful tool to explore the function of essential genes in the parasites’ biology. Two site-specific recombinases, Cre and Flippase recombination enzyme (FLP), have been used successfully to modify the parasites’ genome. Cre and FLP work in a similar fashion via the recognition of a 34-bp sequence, termed locus of X-over in P1 (LoxP) or flippase recognition target (FRT), respectively. The LoxP/FRT sequences flank the DNA of interest (floxed DNA), with the orientation of these sites determining how the genome will be modified by the recombinase. Two sites in the same direction will result in excision of the floxed DNA, while 2 sites in the opposite orientation will result in inversion.

To demonstrate the utility of Cre and FLP in the blood stages, the recombinases were used to remove an integrated drug resistance marker that had previously been used to knock out nonessential genes [4]. These transgenic parasites had the human dihydrofolate reductase (hdhfr) drug resistance cassette, flanked by LoxP or FRT sites, integrated into their genomes, and they were transfected with plasmids containing either Cre or FLP expressed under a tetracycline inducible promoter, such that addition of anhydrotetracycline (aTc) to the parasite culture should result in expression of the recombinase and excision of the drug resistance cassette [4,5]. Cre mediated the removal of the drug marker, but the enzyme was expressed even in the absence of aTc [4]. FLP was shown to be ineffectual, even at its optimal temperature of 30°C, but a subsequent thermostable version (FLPe) proved to be effective at 37°C, the optimal condition for asexual parasite culturing [4,6]. While useful for recycling drug markers, the inability to conditionally regulate these recombinases limited their utility for studying essential proteins in the blood stages.

**Fig 2:**
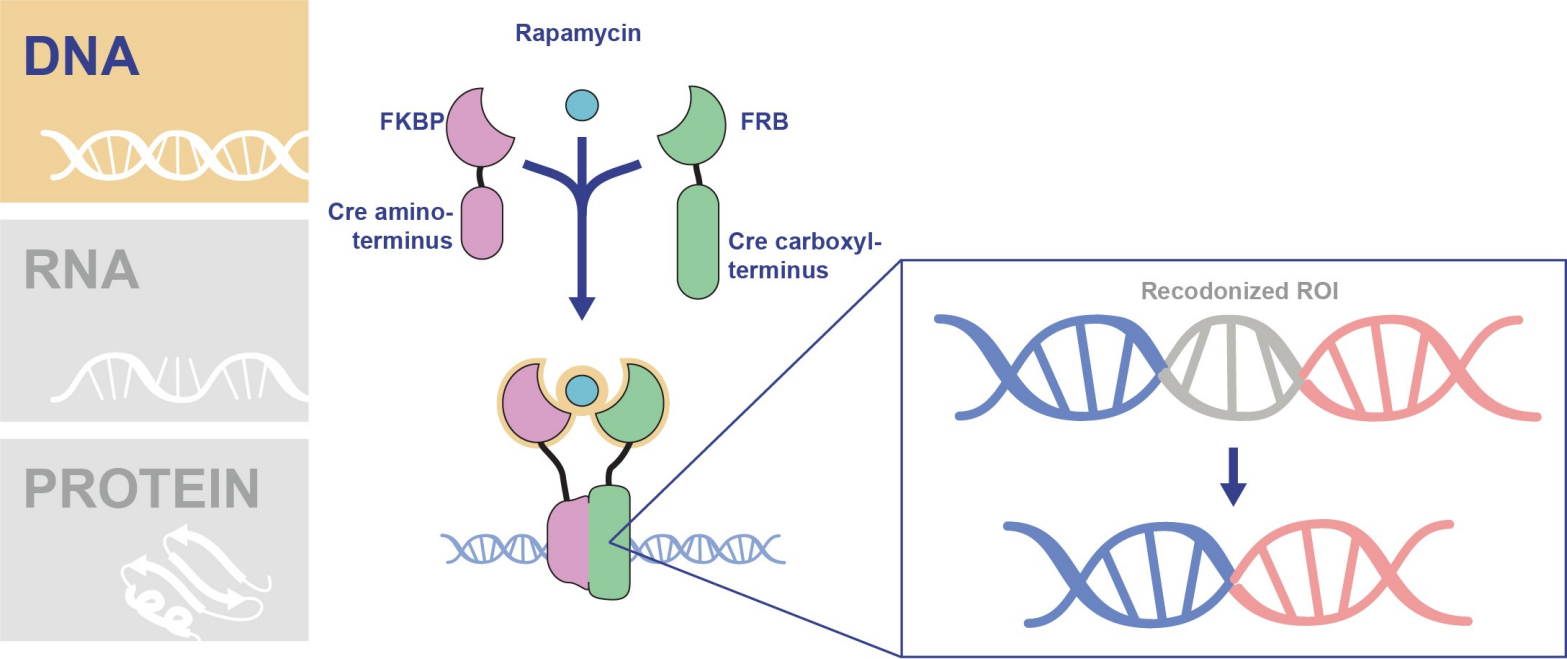
Conditional control of transcription in *P*. *falciparum* using the DiCre system. Transgenic *P*. *falciparum* parasites are created expressing the Cre N-terminus fused to FKBP and the Cre carboxyl terminus fused to FRB. The addition of rapamycin dimerizes the FKBP and FRB proteins which results in the 2 inactive polypeptides joining together to form an active Cre recombinase. The DiCre transgenic parasites are genetically modified to introduce LoxP sites, the sequence recognized by the Cre recombinase, flanking the recodonized DNA sequence of interest. Upon activation of Cre in these parasites using rapamycin, the DNA flanked by LoxP sites is excised, resulting in a conditional knockout. FKBP, FK506 binding protein 12; FRB, FKBP12-rapamycin-binding; LoxP, locus of X-over in P1; ROI, region of interest.

To introduce conditional control over DNA excision, the DiCre system was adapted for use in *P*. *falciparum* blood stages (**Fig 2**) [7]. The DiCre system functions via expression of the Cre recombinase split into 2 separate, inactive proteins [7–10]. Each Cre polypeptide (CreN and CreC terminal) is fused to a rapamycin binding protein—either the FK506 binding protein 12 (FKBP) or the FKBP12-rapamycin-binding (FRB) domain of mTOR [7–10]. Rapamycin induces dimerization of the domains, bringing the Cre polypeptides together to form an active Cre enzyme [7,11,12]. Using this system, Collins and colleagues efficiently excised a floxed hdhfr drug resistance cassette in *P*. *falciparum* only upon rapamycin addition [7]. The system was further adapted for use in *P*. *falciparum* through the creation of an artificial intron containing one of the necessary LoxP sites (LoxPint), allowing for deletion of specific exons or domains, and through development of optimized rapamycin treatment conditions [13,14].

DiCre technology has been used to study parasite egress and invasion [15–18], intracellular signaling [19–21], protein export [22], the essentiality of the artemisinin-resistance marker kelch13 [23], and more. Most conditional systems have been adapted to *P*. *falciparum* strains that are not infectious to mosquitoes, preventing the analysis of protein function in the transmission stages of the parasite. However, a DiCre-expressing line was recently created using the mosquito-infective *P*. *falciparum* NF54 strain in which a DiCre cassette was inserted into the Pfs47 locus, a gene not required for asexual blood stage growth, transmission into *Anopheles stephensi*, or development of the parasite in liver cells [24,25]. This parasite line allowed for conditional gene deletion during the asexual stages in the red blood cell (RBC), during gametocytogenesis, and during intra-hepatic development [24]. This system was also used successfully to interrogate the function of a patatin-like phospholipase (PfPATPL1) in gametogenesis and transmission to the mosquito [26]. Most recently, these DiCre-expressing parasites were used to characterize the entire FIKK kinase family in the asexual stage—identifying essential kinases for growth, host cell remodeling, and virulence [27].

The DiCre system is a fantastic tool to conditionally knock out genes, especially beneficial given that the parasite is haploid in most of its life cycle stages. Furthermore, creative use of the DiCre/LoxPint system allows not only for conditional gene knockouts, but also for the introduction of point mutations into genes. However, the portion of the gene of interest that is flanked by the LoxP sites does require recodonization in the repair plasmid, which can increase the cost of cloning.

### Conditional knockdown of translation

**Fig 3:**
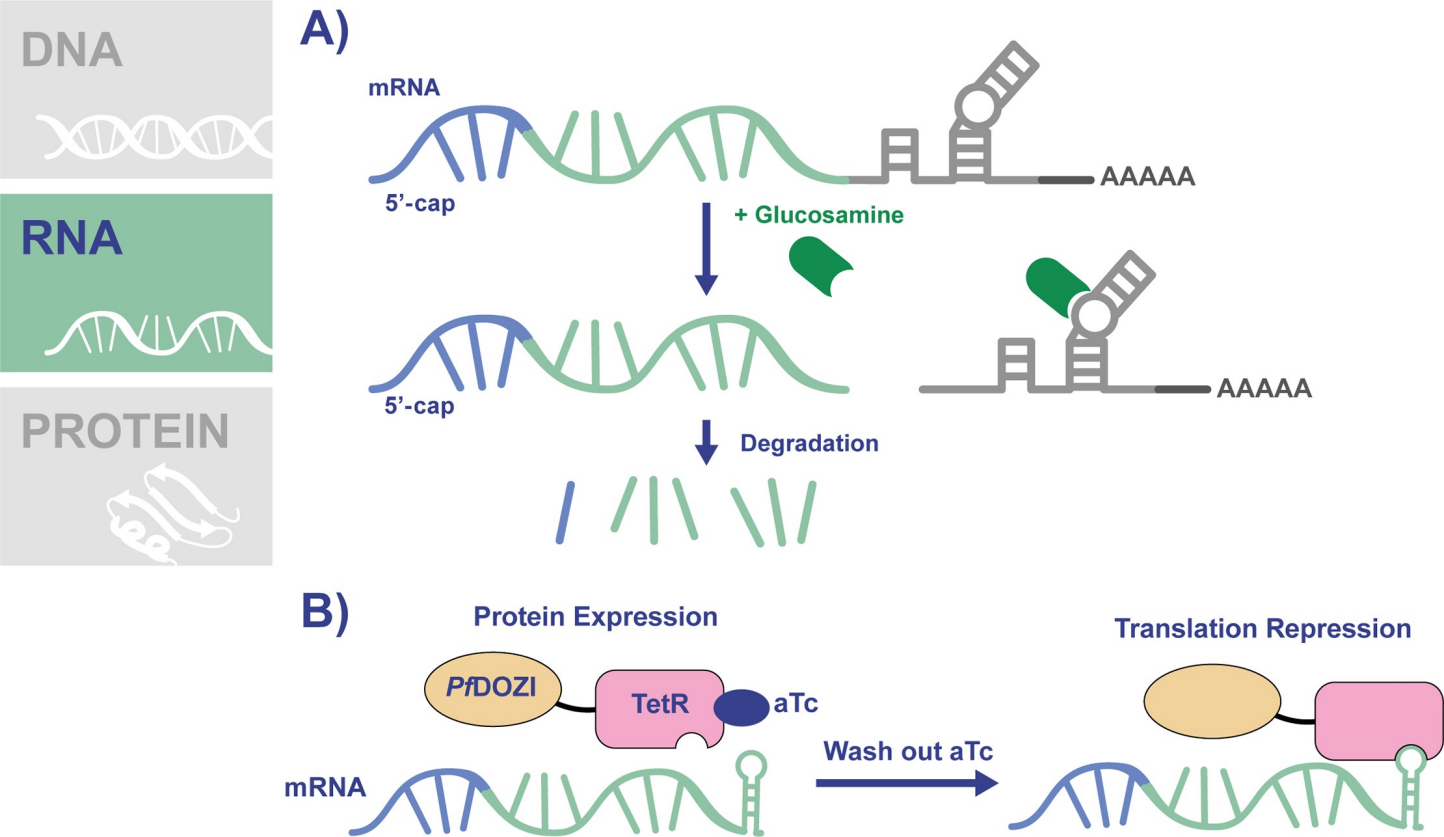
Conditional knockdown of translation using the *glmS* ribozyme or *Pf*DOZI-TetR. **(A)** The *glmS* ribozyme system works by inserting the ribozyme sequence in the parasite genome following the stop codon of the gene of interest. The transcribed mRNA will contain the *glmS* ribozyme (light gray mRNA), which is activated by glucosamaine-6-phosphate to cleave its associated RNA, resulting in transcript instability and degradation. **(B)** The TetR-DOZI system works by the insertion of 10 copies of the TetR aptamer sequence following the stop codon of the gene of interest. A PfDOZI-TetR fusion protein is expressed in these parasites. TetR recognizes and binds to the RNA aptamers (green hairpin), and *Pf*DOZI directs the mRNA to sites of translation repression. The addition of aTc inhibits the binding of *Pf*DOZI-TetR to the mRNA. Therefore, translation of the protein of interest can be regulated by the addition or removal of aTc. aTc, anhydrotetracycline.

Induction of protein knockdown by targeting mRNA translation has been a useful strategy for studying protein function across many organisms, with RNA interference (RNAi) methods widely used, including in some protozoans [28]. This method involves the introduction of double-stranded RNA into cells, which is processed and then binds homologous mRNA within the cell, leading to transcript degradation. Unfortunately, *Plasmodium* parasites lack a functional RNAi pathway, leading to the development of other RNA-based knockdown systems for use in the parasites [29]. One such example is the *glmS* knockdown system, which uses an activatable ribozyme from gram-positive bacteria [30–32]. The *glmS* ribozyme sequence is inserted into the gene of interest after the stop codon so that the transcribed mRNA contains the ribozyme, which is activated by glucosamine-6-phosphate to cleave its associated mRNA, leading to transcript instability and degradation (**Fig 3A**). Modification of the parasite genome to introduce the ribozyme is relatively uncomplicated, with a common approach being the simultaneous introduction of an epitope tag sequence at the end of the ORF, a stop codon, then the ribozyme sequence. To activate the ribozyme in *P*. *falciparum* parasites, culture medium is supplemented with glucosamine, which is converted to glucosamine-6-phosphate by the parasite. An advantage of this system is the availability of an inactive “M9” version of the ribozyme containing a single point mutation that renders it unable to cleave mRNA [32]. Parasite lines expressing mRNA encoding the M9 version serve as a control to their *glmS*-expressing counterparts.

The ribozyme has been used to dissect diverse aspects of *P*. *falciparum* asexual biology, including hemoglobin degradation and nutrient uptake [33–35], protein trafficking and export [36–38], plastid function [39,40], egress and invasion [41–43], and more. One concern associated with the *glmS* system is whether the level of knockdown achieved will result in an observable phenotype. For example, the use of the *glmS* system to interrogate the essentiality of the proteases *Pf*PMV and *Pf*ClpP showed no growth defects when the proteases were knocked down [39,44]. However, both proteases were subsequently shown to be essential via other methods [22,40,45]. These observations suggest that while the *glmS* system has proved invaluable in studying parasite biology, it may have limited utility for establishing the essentiality of some proteins with high expression or low turnover.

The *Pf*DOZI-TetR is another conditional system for controlling protein expression via translation (**Fig 3B**) [46,47]. In this system, 10 copies of a TetR-binding aptamer sequence are inserted following the stop codon of the gene of interest [48]. The aptamers in the mRNA are recognized by the TetR protein, which is fused to *Pf*DOZI, a protein that localizes to mRNA sequestration sites known as P-bodies. Binding of *Pf*DOZ-TetRI to the mRNA leads to its relocalization to P-bodies and repression of translation. Introduction of this entire system into the parasite genome can be achieved using CRISPR/Cas-9 and a linearized repair template that contains homology regions to the gene of interest, an epitope tag, the 10x aptamer sequences, a cassette to express the *Pf*DOZI-TetR regulatory fusion protein, and a drug selection marker [46]. A single aptamer in the 5′ UTR of the mRNA may also be included in order to increase the level of repression [45,46]. The TetR-aptamer interaction is inhibited by the presence of aTc. Therefore, parasite culturing in the presence of aTc sustains continuous protein expression, while conditional protein knockdown is achieved by removing aTc.

Although its mode of function and use is more complex compared to the *glmS* system, the PfDOZI-TetR system has become increasingly popular in the study of asexual parasite biology. For example, the system has been used to understand the roles of proteins in egress and invasion [41,49,50], parasite membrane homeostasis and vacuole morphology [51,52], nutrient acquisition [53,54], and apicoplast biology [2,39,40,55,56]. Regarding the latter, Tang and colleagues used the *Pf*DOZI-TetR system to validate results from a forward genetic screen aimed at uncovering genes essential for apicoplast biogenesis. Their work demonstrates the importance of having conditional knockdown tools for researching druggable pathways in the parasite.

The *glmS* and *Pf*DOZI-TetR systems are both mRNA-targeting knockdown systems that have been used to answer pressing biological questions regarding the *P*. *falciparum* blood stages. An attractive feature of the *glmS* system is its ease of use (simple molecular cloning and addition of a molecule to induce knockdown). In our experience, the *Pf*DOZI-TetR system achieved a greater knockdown than the *glmS* system when used to study the apicoplast resident protease *Pf*ClpP, demonstrating that the same protein may respond differently to different conditional systems [39,40]. In contrast to the relatively easier molecular cloning involved in the generation of *glmS* mutant parasites, the plasmids used for generating *Pf*DOZI-TetR mutants are much larger and prone to recombination, although growing bacteria containing the plasmids at 30°C seems to help with this particular issue. Additionally, performing the cloning steps with a linear plasmid has also been reported to make the cloning easier [57]. It has been reported (and we have observed) that the loss of aptamer copies can also occur in clonal parasites that originally possessed all 10 aptamer copies, leading to loss of knockdown efficiency. Therefore, a newer, optimized version of the TetR system has been recently developed that is less prone to loss of the aptamer copies via recombination [58]. However, one caveat of the *glmS* system is that the drug used to induce knockdown, glucosamine, can be toxic to asexual parasites at certain concentrations [44]. Both systems continue to be widely used for researching *P*. *falciparum* biology, and we expect the ability to use both systems simultaneously will prove to be powerful [40,41].

### Conditional knockdown of protein function

**Fig 4:**
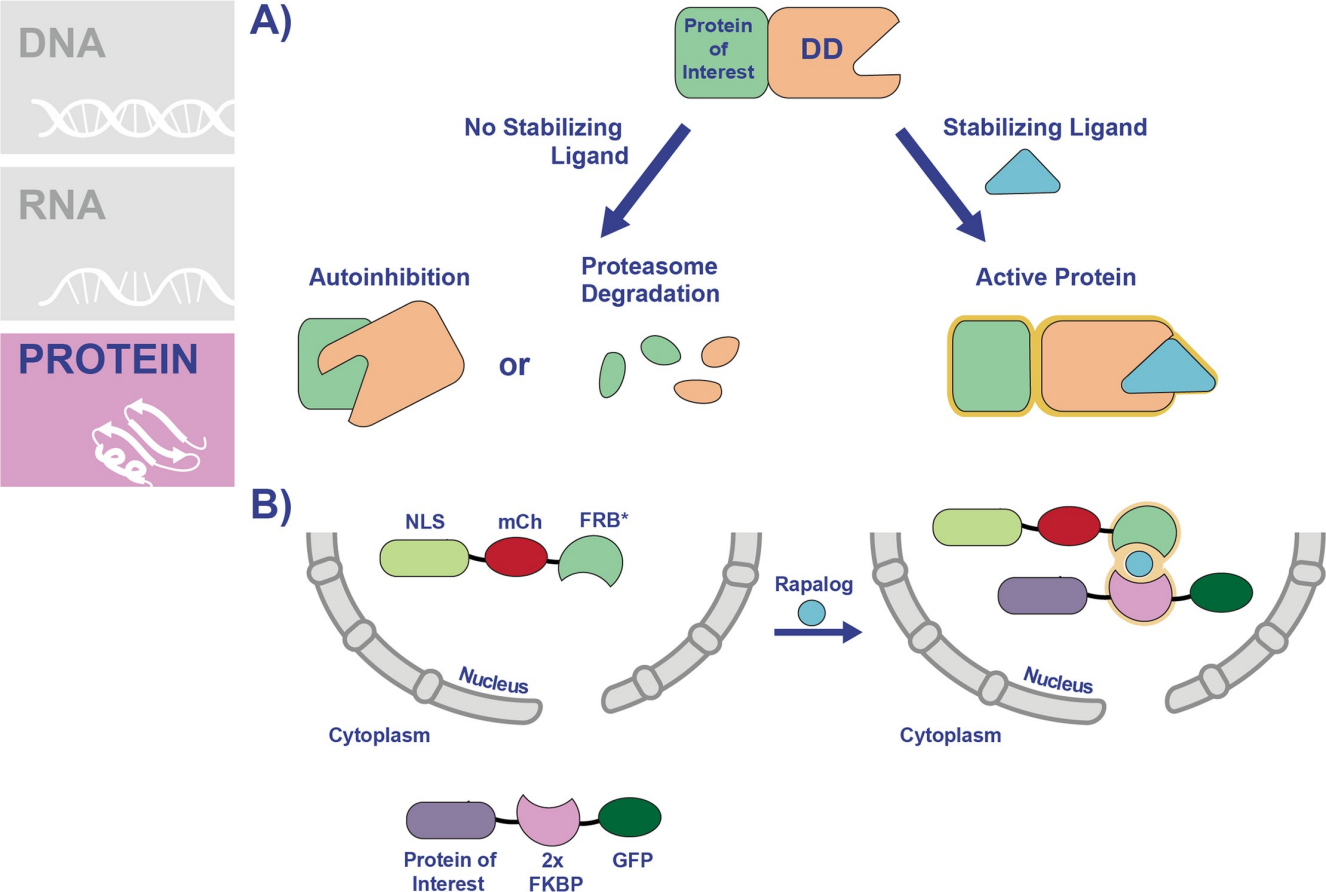
Conditional knockdown of protein function using degradation or KS systems. **(A)** The FKBP-DD and DD systems work using a similar mechanism, thus a combined schematic of how these systems work is shown. For simplicity, both the FKBP-DD and DD are depicted and referred to as DD. The sequence encoding the DD is inserted into the genome in frame immediately following the gene of interest. The DD contains point mutations which render the protein domain unstable, causing the protein to be recognized for degradation by the proteasome. In the case of chaperone proteins appended to the DD, the unfolded domain is recognized by the chaperone, which results in autoinhibition. The DD is stabilized through the addition of a ligand (TMP for DD and Shld1 for FKBP-DD). Addition of the ligand stabilizes the DD to prevent its degradation by the proteasome or autoinhibition. **(B)** The KS system allows for the conditional mislocalization of proteins to inhibit their functions. In this system, the protein of interest is fused to 2 copies of the FKBP domain in a parasite line that expresses an FRB* “mislocalizer” fusion protein, engineered to localize to the nucleus or plasma membrane. Addition of rapamycin (or rapalog) dimerizes the FKBP/FRB* domains, resulting in the relocalization of the protein of interest to one of these sites. DD, destabilization domain; FKBP, FK506 binding protein 12; FKBP-DD, FK506 binding protein destabilization domain; FRB, FKBP12-rapamycin-binding; GFP, green fluorescent protein; KS, knock sideways; mCh, mCherry; NLS, nuclear localization signal; TMP, trimethoprim.

Unlike the methods that target transcription or translation, knockdown at the protein level is advantageous because the inhibition is applied directly to the protein of interest. Therefore, these fast-acting systems are good for studying rapid processes such as protein trafficking or cell cycle. The FK506 binding protein destabilization domain (FKBP-DD) was one of the first systems adapted to modulate protein levels in *P*. *falciparum* (**Fig 4A**) [59]. In this method, the FKBP-DD contains destabilizing mutations that induce misfolding of the domain in the absence of the stabilizing ligand Shld1 [60]. FKBP-DD can be appended to the N-terminus or carboxyl terminus of the protein of interest, inducing its degradation via the proteasome [59]. However, upon the addition of Shld1, the domain is stabilized, and the protein is not targeted for degradation. Initially, the FKBP-DD was used to study parasite proteases, and the system was further refined by including an epitope tag to more easily monitor protein degradation [59,61]. Subsequently, the FKBP-DD system has found widespread use in the study of disparate parasite pathways, some of which are temporally regulated. These include schizont segmentation [62], nuclear division and DNA replication [63], and parasite egress from the host RBC [64–66].

In 2011, a similar protein degradation system was implemented in *P*. *falciparum* using the dihydrofolate reductase (DHFR) domain from *Escherichia coli* [67]. This domain, termed the DHFR degradation domain (DDD), also contains mutations that render it unfolded [67,68]. The unfolded domain is targeted for degradation in the absence of the small drug molecule trimethoprim (TMP). The system was first used to demonstrate the ability to degrade yellow fluorescent protein tagged with the DDD as well as the essentiality of the proteasome subunit, RPN6, in *P*. *falciparum* asexual parasites [67]. Notably, the TMP concentrations required to stabilize the domain are toxic to the parasite; therefore, DDD-mediated protein knockdowns are performed in a TMP-resistant parasite line that have the human DHFR cassette integrated into the nonessential gene, *plasmepsin I*. Interestingly, DDD tagging of a cytoplasmic chaperone (HSP110) revealed that in the absence of TMP, chaperones are not targeted for degradation. Rather, inhibition was achieved through the chaperone binding to the unfolded domain, thereby preventing interactions with client proteins [69]. Therefore, this system has been widely used to study chaperone function in the asexual stages of *P*. *falciparum* [36,39,70,71]. However, the utility of the DDD system has also been demonstrated for a variety of other proteins, including those involved in nutrient uptake (RhopH2 and RhopH3) and RBC invasion (RhopH3) [34]. Furthermore, it has been used to identify a protein (PfVAP1) that is important for binding of the virulence factor PfEMP1 to CD36, a microvascular receptor [72].

There are disadvantages associated with both the FKBP-DD and DDD systems. Both systems require appending domains to the N-terminus or carboxyl terminus of the protein, which could potentially alter protein function or localization. Additionally, both systems require the stabilizing ligand, be it Shld1 or TMP, to be constantly supplied to the parasite to maintain stable expression of the protein of interest. Shld1 can be toxic to asexual parasite growth at high concentrations, and TMP is toxic to parasites unless used in a parasite line expressing the hDHFR [73]. With both systems, studying membrane proteins or proteins located throughout the parasite’s secretory pathway may be difficult, as the protein may not have access to the proteasome for degradation or be able to bind, in the case of the DDD, the unfolded domain [74].

Another option which does not utilize an unfolded domain is the knock sideways (KS) system, which relies on subcellular relocalization of the protein of interest to prevent its function. In *P*. *falciparum*, a KS system was recently established using the rapamycin-dimerizable domains FKBP and FRB* [23]. In this system, the protein of interest is fused to 2 copies of the FKBP domain in a parasite line that expresses a “mislocalizer” fusion protein (**Fig 4B**). The mislocalizer consists of the FRB* domain engineered to localize to the nucleus or plasma membrane, so that the addition of rapamycin results in the relocalization of the protein of interest to one of these sites. KS was used to show for the first time that Kelch 13 (K13), a protein involved in artemisinin resistance, is important for asexual replication [23]. A subsequent KS study showed that K13 is part of an endocytic pathway that imports hemoglobin into the parasite and identified additional K13-interacting proteins as part of this pathway [75]. Another study, also concerning endocytosis of hemoglobin, showed that PfVPS45 is required for the process using KS [76]. KS likely has similar limitations as the previously discussed degradation-based systems. Namely, the protein of interest must be amenable to fusion with large domains, and it must have access to the mislocalizer protein (i.e., limited utility might be expected with exported proteins, unless a new mislocalizer protein is expressed and exported as well).

### Choosing a system for your gene of interest

There are several factors that need to be considered when choosing a specific conditional system to study a gene of interest. Because we have several systems that work for conditional expression of proteins in *P*. *falciparum*, the rubric we (and others) have used as well as our collective experience with these tools allow us to offer a pathway for selecting a specific system. The first criteria to consider is the putative function of the gene. In our (and the field’s) experience, proteins with enzymatic activity require a greater degree of knockdown before a phenotype is observed [22,39,40,44,45,63]. This discrepancy is due to the fact that often, normal parasite growth in culture may only require 5% to 10% of enzyme expression. Therefore, in these cases, the knockdown has to down-regulate protein expression >95% before a phenotype is observed. For example, the PEXEL protease PMV was first identified as a critical factor required for protein export a decade ago [74,77]. However, the knockdown achieved by the *glmS* ribozyme-based system was unable to prove the essentiality of PMV for the asexual life cycle, even though PMV expression was downregulated by 4- to 5-fold [44]. Genetic evidence for essentiality of PMV was obtained recently using diCre-based conditional knockout and *tetR* aptamer-based conditional knockdown, with both systems inducing >99% inhibition of protein expression [22,45].

Another rubric to consider is the putative biological process where the gene of interest functions. This can be critical because if the kinetics of knockdown is slower than the biological process, interpreting the phenotype can be confounded. For example, the endocytic pathway in *P*. *falciparum* was deciphered elegantly using the KS approach that functions in the same time frame as endocytosis and thus, allowed the functional dissection of the *kelch13* gene as well as its associated partners [75].

Another factor one should consider is the cellular localization of the gene or whether the gene has transmembrane domains. Conditional systems that depend upon a second factor to function, such as the proteasome for the DD or the mislocalizer for KS, may only work optimally in the cellular compartment where the second factor is present. Should this be a concern, the diCre-based conditional knockout or the conditional *glmS* ribozyme or *tetR* aptamer systems are the best options.

However, given that a large proportion of *Plasmodium* genes are of unknown function, a rational choice is not always available. In these cases, one selection criteria could be the life cycle stage under study, i.e., if the gene is expressed during the insect stages of the life cycle, then one could choose a system that works in all stages of the parasite life cycle, or a system where the effector molecule can be delivered into the desired mosquito compartment, i.e., the hemocoel or the salivary glands to target oocysts and sporozoites [24]. The diCre-based conditional knockout method shows promise in these cases because it has been demonstrated to work in the mosquito stages, and because it results in gene deletion, deciphering the essentiality of the gene is more straightforward.

## Conclusions

The last decade has shown remarkable progress toward understanding the biology of *P*. *falciparum* parasites. The development and utilization of conditional knockdown systems have allowed researchers to probe the essential functions of proteins that support parasite growth, as well as explore the roles of proteins that are nonessential in asexual culture but may nonetheless prove important in other life stages and in pathogenesis. In turn, these proteins and their biological pathways may represent new avenues for the development of antimalarial drugs. We have discussed some popular systems here, but others have been developed and are being used to answer pressing questions, including a conditional localization approach for apicoplast proteins [78] and the auxin-induced protein degradation system [79,80]. Additionally, the establishment of CRISPR/Cas-9 genome editing and development of selection-linked integration into the field of *P*. *falciparum* research has rendered the parasites more genetically tractable [23,81,82]. Therefore, as new conditional systems are developed, the historical difficulty in editing the parasite genome will not hinder their introduction into *P*. *falciparum* research. Of particular interest are the new developments regarding dead Cas-9—used to physically occupy targeted DNA sequences and prevent their transcription, and which has been used to target histone-modifying proteins to genes in *Plasmodium—*and RNA-degrading Cas-13s, which have not yet been applied to study *Plasmodium* [83–86]. The RNA-targeting Cas-13 RNA nucleases are particularly promising because just like RNAi, they only require a short gRNA (approximately 20 bp) for knocking down the gene of interest. This precludes the need for cloning large homology regions for targeting the gene of interest via homology directed repair, which often takes several weeks because of difficulties in cloning the AT-rich genome of *P*. *falciparum*. The ability to use short gRNAs for knocking down gene expression also promises the ability to multiplex knockdown of several genes at once that can prove useful in understanding functional redundancy. As these cutting-edge technologies continue to be adapted to *Plasmodium—*and like the Cre system, further refined so that researchers can conditionally control their function—these indispensable tools will accelerate our understanding of *Plasmodium* biology in all stages of its life cycle.

Much of the work that has been performed using conditional systems has been done using asexual stage parasites. However, extraerythrocytic stages are critical points for therapeutic intervention, and application of conditional systems outside of asexual replication will allow researchers to answer critical questions about the biology of these stages. Because the systems outlined here work in the asexual stages from which the gametocytes are directly derived, we anticipate that many of them will be amenable for studying proteins that perform essential functions in gametocytes. Indeed, the *glmS* ribozyme has been used for conditional knockdown of proteins critical for gametocyte development [87,88]. DD-based knockdown at the protein level has also been used to characterize proteins in gametocytogenesis [70,89–91]. More recently, the DiCre system has been used to study proteins during intraerythrocytic growth, gametocytogenesis, and liver stage development in *P*. *falciparum* [24,26]. DiCre has been used in *Plasmodium berghei* to excise parasite genes in the mosquito midgut by feeding the mosquito rapamycin [92]. In theory, this could also be done using *P*. *falciparum*. Knockdown of parasite genes could possibly be achieved in the mosquito vector, for the glmS and TetR-DOZI systems, by providing the effector molecules in the blood meal, sugar meal, and/or injection into the hemolymph. For liver stages, sporozoites could be incubated with the molecules prior to infection in hepatocytes. The same could be done with TMP or Shld1 for the DD systems, except here the drug would be removed at various stages of parasite development and not added. However, additional research is needed to determine the functionality of these conditional systems in the transmission stages of the parasite, both during development in the mosquito and transmission to the vertebrate host.

**Key Learning Points**

- There are several excellent tools available to study *Plasmodium falciparum* protein function.
- Available systems can regulate protein expression by targeting DNA, RNA, or the protein.
- Several conditional knockdown (and knockout) systems are being tested in the exoerythrocytic stages.
- Choosing a specific system depends on the protein of interest, stage under study, and putative biological function.
- Some systems can be adapted to study protein function during parasite development in the mosquito and transmission.

**Top Five Papers**

1. Ghorbal M, Gorman M, Macpherson CR, Martins RM, Scherf A, Lopez-Rubio J-J. Genome editing in the human malaria parasite *Plasmodium falciparum* using the CRISPR-Cas9 system. Nat Biotechnol. 2014 Aug 1;32(8):819–21.
2. Wagner JC, Platt RJ, Goldfless SJ, Zhang F, Niles JC. Efficient CRISPR-Cas9–mediated genome editing in *Plasmodium falciparum*. Nat Methods. 2014 Sep;11(9):915–8.
3. Prommana P, Uthaipibull C, Wongsombat C, Kamchonwongpaisan S, Yuthavong Y, Knuepfer E, et al. Inducible Knockdown of *Plasmodium* Gene Expression Using the glmS Ribozyme. Craig AG, editor. PLoS ONE. 2013 Aug 30;8(8):e73783.
4. Ganesan SM, Falla A, Goldfless SJ, Nasamu AS, Niles JC. Synthetic RNA–protein modules integrated with native translation mechanisms to control gene expression in malaria parasites. Nat Commun. 2016 Apr;7(1):10727.
5. Jones ML, Das S, Belda H, Collins CR, Blackman MJ, Treeck M. A versatile strategy for rapid conditional genome engineering using loxP sites in a small synthetic intron in Plasmodium falciparum. Sci Rep. 2016 Feb 19;6:21800.
